# Supplementary material for: Role of Palliative Care in Onco-Hematology Retrospective Observational Cohort Study in Deceased In-Hospital Patients with SACT at the End of Life: Experience with Real-World Data from a Cancer Monographic Institution
Source: Cancers (Basel). 2025 Oct 28;17(21):3467. doi: 10.3390/cancers17213467 (PMC12608604; doi:10.3390/cancers17213467)
Supplement: Supplementary file 1 [file cancers-17-03467-s001.zip › cancers-3880864-supplementary.pdf]

Supplementary Materials:

Table S1. In hospital mortality with SACT ≤ 30d. Comparison table between periods: First period "pre study of symptoms" (2017-2019) / Second period "study of symptoms" (2020-2023).

Summary descriptive table by groups of Symptoms Period			
	**FIRST PERIOD (2017-2019)**	**SECOND PERIOD (2020-2023)**	**p-value**
	**N=1055 (39%)**	**N=1681 (61%)**	
**CENTER:**			**0.587**
1	322 (30.5%)	491 (29.2%)	
2	290 (27.5%)	491 (29.2%)	
3	443 (42.0%)	697 (41.5%)	
**PATHOLOGY GROUP:**			**0.522**
Hematological Neoplasia (HN)	267 (25.3%)	445 (26.5%)	
Solid Tumor (ST)	788 (74.7%)	1235 (73.5%)	
**SEX:**			**0.032**
Female	384 (36.4%)	682 (40.6%)	
Male	671 (63.6%)	999 (59.4%)	
**CURRENT AGE**	**63.8 (12.3)**	**65.5 (12.3)**	**<0.001**
**AGE 65y:**			**0.001**
<=65y	537 (50.9%)	741 (44.1%)	
>65y	518 (49.1%)	940 (55.9%)	
**ECOG VALUE:**			**0.012**
0	29 (4.37%)	70 (7.98%)	
1	293 (44.1%)	390 (44.5%)	
2	283 (42.6%)	321 (36.6%)	
3	53 (7.98%)	86 (9.81%)	
4	6 (0.90%)	10 (1.14%)	
**PS RECORDED**			**<0.001**
No Documented (N/D)	192 (18.2%)	51 (3.03%)	
No	127 (12.0%)	704 (41.9%)	
Yes	736 (69.8%)	926 (55.1%)	
**BMI CATEGORIES:**			**<0.001**
Underweight	38 (3.60%)	41 (2.44%)	
Normal weight	429 (40.7%)	362 (21.5%)	
Overweight	190 (18.0%)	169 (10.1%)	
Obesity	73 (6.92%)	74 (4.40%)	
Morbid obesity	4 (0.38%)	6 (0.36%)	
N/D	321 (30.4%)	1029 (61.2%)	
**Hb (gr/dl)**	11.1 (5.66)	11.5 (8.19)	**0.091**
**PLATELETS (x10E9)**	No recorded	235 (153)	**.**
**ALBUMIN (gr/l)**	33.2 (6.92)	33.7 (6.86)	**0.162**
**LDH (ukat/l)**	No recorded	512 (712)	**.**
**COMORBIDITIS:**			**0.001**
N/D	11 (1.05%)	2 (0.12%)	
No	189 (18.0%)	261 (15.7%)	
Yes	852 (81.0%)	1396 (84.1%)	
**HOW MANY COMORBIDITIES?**<br>-mean<br>-median	 2.98 (1.76)<br>3	 3.36 (2.01)<br>3	 **<0.001**

**COMORB. Second primary**	**194 (18.4%)**	**323 (19.2%)**	**0.626**
**TUMOR TYPUS:**			**0.500**
Breast	69 (6.55%)	118 (7.03%)	
CNS	33 (3.13%)	30 (1.79%)	
Colon	45 (4.27%)	77 (4.59%)	
CUO (origin unknown)	11 (1.04%)	13 (0.77%)	
Esophagus	19 (1.80%)	25 (1.49%)	
Gastric	41 (3.89%)	47 (2.80%)	
Gynecological	44 (4.18%)	84 (5.00%)	
Head and Neck	50 (4.75%)	74 (4.41%)	
Hepatobiliary	10 (0.95%)	13 (0.77%)	
Hodking	7 (0.66%)	8 (0.48%)	
Leukemia	101 (9.59%)	164 (9.77%)	
Lung	273 (25.9%)	479 (28.5%)	
Melanoma	21 (1.99%)	27 (1.61%)	
Myeloma	51 (4.84%)	67 (3.99%)	
Neuroendocrine Tumor	6 (0.57%)	8 (0.48%)	
NHL	80 (7.60%)	145 (8.64%)	
Pancreas	55 (5.22%)	75 (4.47%)	
Prostate	12 (1.14%)	30 (1.79%)	
Rectal	10 (0.95%)	13 (0.77%)	
Sarcoma	21 (1.99%)	22 (1.31%)	
Urological	53 (5.03%)	82 (4.88%)	
Others	41 (3.89%)	78 (4.65%)	
**INITIAL STAGE:**			**.**
0/In Situ	1 (0.12%)	3 (0.21%)	
I	24 (2.83%)	49 (3.44%)	
II	45 (5.30%)	88 (6.18%)	
III	168 (19.8%)	305 (21.4%)	
IV	451 (53.1%)	773 (54.3%)	
Not apply (Leuk./ CNS / …)	160 (18.8%)	205 (14.4%)	
**THERAPEUTIC SCHEME:**			**.**
Scheme Change	268 (25.4%)	369 (22.0%)	
Continued	579 (54.9%)	900 (53.5%)	
Debut	189 (17.9%)	351 (20.9%)	
N/D	19 (1.8%)	61 (3.64%)	
**SACT-Death interval:**			**0.364**
SACT_15-30d	630 (59.7%)	973 (57.9%)	
SACT<=14d	425 (40.3%)	708 (42.1%)	
**DAYS OF STAY (last admission):**			**0.720**
<=14 dies	765 (72.5%)	1207 (71.8%)	
>14 dies	290 (27.5%)	474 (28.2%)	
**AGGRESSIVENESS SCALE**			**0.923**
0	192 (18.2%)	306 (18.2%)	
1	385 (36.5%)	603 (35.9%)	
2	311 (29.5%)	483 (28.7%)	
3	132 (12.5%)	218 (13.0%)	
4	31 (2.94%)	64 (3.81%)	
5	4 (0.38%)	6 (0.36%)	

6	0 (0.00%)	1 (0.06%)	
**PLACE SACT ADMINISTRATION:**			**0.600**
Ambulatory	150 (14.2%)	227 (13.7%)	
Day Hospital	641 (60.8%)	986 (59.4%)	
Inhospital room	262 (24.9%)	444 (26.7%)	
N/D	1 (0.09%)	4 (0.24%)	
**PLACE OF DEATH:**			.
Onco-hematologic room	670 (63.7%)	1051 (62.7%)	
PC room	229 (21.8%)	311 (18.6%)	
H. Reference Acute	34 (3.24%)	100 (5.97%)	
ICU	101 (9.61%)	184 (11.0%)	
Emergencies room	13 (1.24%)	25 (1.49%)	
N/D	4 (0.38%)	4 (0.24%)	
CAUSE OF DEATH:			.
Intercurrent	62 (5.88%)	91 (5.53%)	
Intercurrent-CoviD	0 (0.00%)	25 (1.52%)	
Mixed	136 (12.9%)	193 (11.7%)	
Mixed-CoviD	0 (0.00%)	26 (1.58%)	
Related to treatment	71 (6.73%)	23 (1.40%)	
Related to current tumor	780 (73.9%)	1282 (77.9%)	
Second primary neoplasia	6 (0.57%)	5 (0.30%)	
**ADV ADVANCED DISEASE:**			**0.007**
Yes	798 (75.6%)	1308 (77.8%)	
No	210 (19.9%)	268 (15.9%)	
N/D	47 (4.45%)	105 (6.25%)	

Legend: Categorical variables: Frequencies (Percentage, %); Numerical normal distributed variables: Mean (Standard Deviation); Numerical non-normal distributed variables: Median [25th; 75th percentiles]. If p-value < 0.05 it means that differences between groups in means or proportions are statistically significant at 5% of significance level.

Notes: PS: performance status (combination of ECOG and Karnofsky variables); N/D: not documented; BMI: body mass index; Hb: hemoglobin; LDH: lactodehydrogenase; CUO: carcinoma of unknown origin; CNS: central nervous system; NHL: non-Hodgkin lymphoma; SACT: systemic anticancer therapy (without differentiating chemotherapy from targeted therapies & except hormone therapy and vaccines); Aggressiveness scale: adapted to that described by Hui; PC: palliative care. The causes of death include new categories: mixed that involves therapy and tumor (especially in hematology), and in the second period, CoviD was added to intercurrent and mixed deaths, not as a sole cause. (*): This percentage is not the internationally defined indicator, it is only the proportion of cases within the subgroup of deaths SACT<30d.

Figure S1. Distribution of patients in Palliative Care Program (PCP) or not, by type of tumor pathology (2020-2023).

Plot

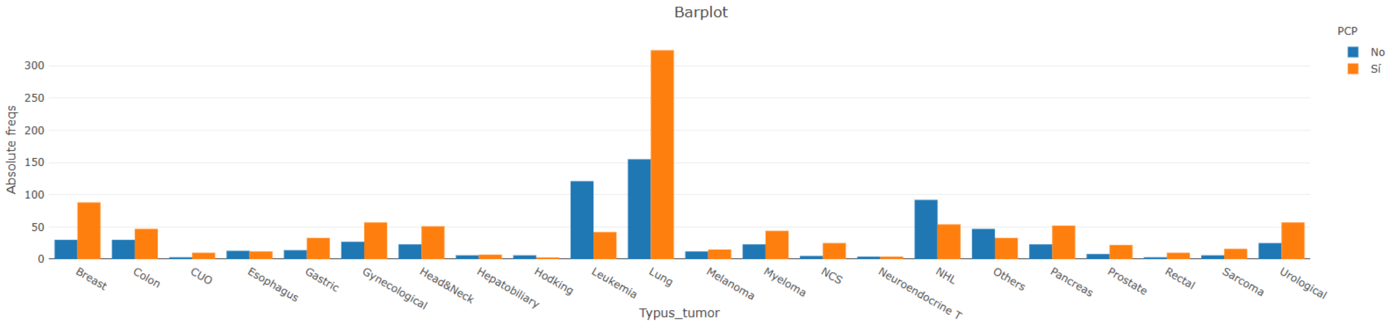

Statistics

PCP	Breast	Colon	CUO	Esophagus	Gastric	Gynecological	Head&Neck	Hepatobiliary	Hodking	Leukemia	Lung	Melanoma	Myeloma	NCS	Neuroendocrine T	NHL	Others	Pancreas	Prostate	Rectal	Sarcoma	Urological
No	30	30	3	13	14	27	23	6	6	121	155	12	23	5	4	92	47	23	8	3	6	25
Sí	88	47	10	12	33	57	51	7	2	42	324	15	44	25	4	54	33	52	22	10	16	57

Note: The types of tumors included in PCP are in orange and in blue those that are not included in PCP

Figure S2. Variations in "pain" registry depending on the type of tumor and PCP groups by type of tumor pathology (in relative percentages).

a) Registry of Pain & tumor types &PCP=No

Plot

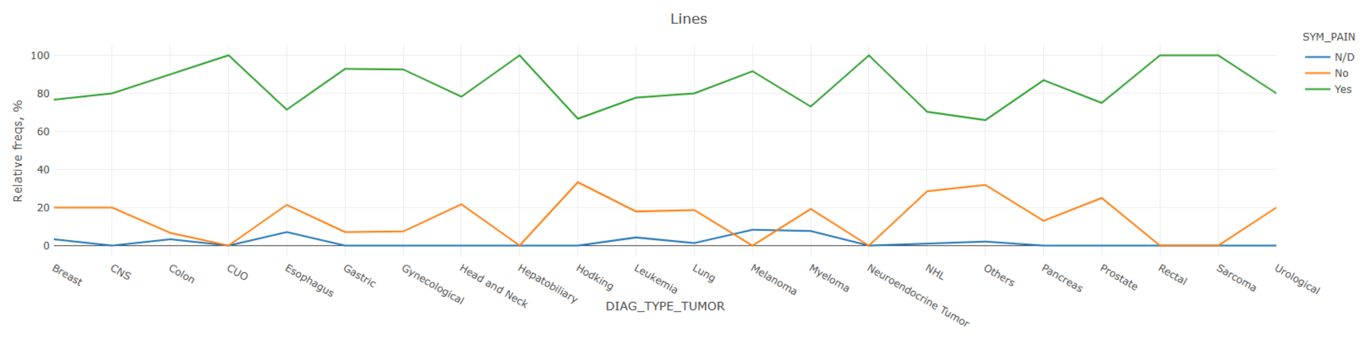

Statistics

SYM_PAIN	Breast	CNS	Colon	CUO	Esophagus	Gastric	Gynecological	Head and Neck	Hepatobiliary	Hodking	Leukemia	Lung	Melanoma	Myeloma	Neuroendocrine Tumor	NHL	Others	Pancreas	Prostate	Rectal	Sarcoma	Urological
N/D	3.3	0	3.3	0	7.1	0	0	0	0	0	4.3	1.3	8.3	7.7	0	1.1	2.1	0	0	0	0	0
No	20	20	6.7	0	21.4	7.1	7.4	21.7	0	33.3	17.9	18.7	0	19.2	0	28.6	31.9	13	25	0	0	20
Yes	76.7	80	90	100	71.4	92.9	92.6	78.3	100	66.7	77.8	80	91.7	73.1	100	70.3	66	87	75	100	100	80

b) Registry of Pain & tumor types & PCP=Yes

Plot

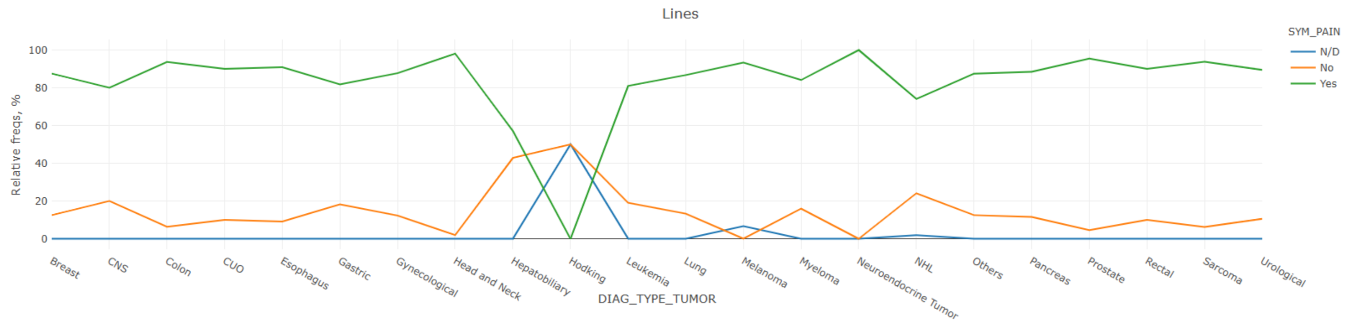

Statistics

SYM_PAIN	Breast	CNS	Colon	CUO	Esophagus	Gastric	Gynecological	Head and Neck	Hepatobiliary	Hodking	Leukemia	Lung	Melanoma	Myeloma	Neuroendocrine Tumor	NHL	Others	Pancreas	Prostate	Rectal	Sarcoma	Urological
N/D	0	0	0	0	0	0	0	0	0	50	0	0	6.7	0	0	1.9	0	0	0	0	0	0
No	12.5	20	6.4	10	9.1	18.2	12.3	2	42.9	50	19	13.3	0	15.9	0	24.1	12.5	11.5	4.5	10	6.2	10.5
Yes	87.5	80	93.6	90	90.9	81.8	87.7	98	57.1	0	81	86.7	93.3	84.1	100	74.1	87.5	88.5	95.5	90	93.8	89.5

Note: N/D (in blue): not registered data; Yes (in green): registered data and the symptoms are positive; No (in orange): registered data and the symptoms are negative. Pain in a) was registered in 97.24%, and register was positive in 75.6%; in b) was registered in 99.66%, and register was positive in 85.6%. Statistical difference between groups a) & b): $p<0.001$ (see table 1)

In b) "Yes-PCP" group (patients included in the palliative care program), the overall registration percentage is higher than in a) "No-PCP" group, and the positive registrations are higher too. In the case of Hodgkin's disease, there are only two cases, which is why the curve produces an abrupt inflection.

Figure S3. Variations in the "anxiety" registry depending on the type of tumor and PCP groups.

a) Registry of Anxiety & tumor types & PCP=No

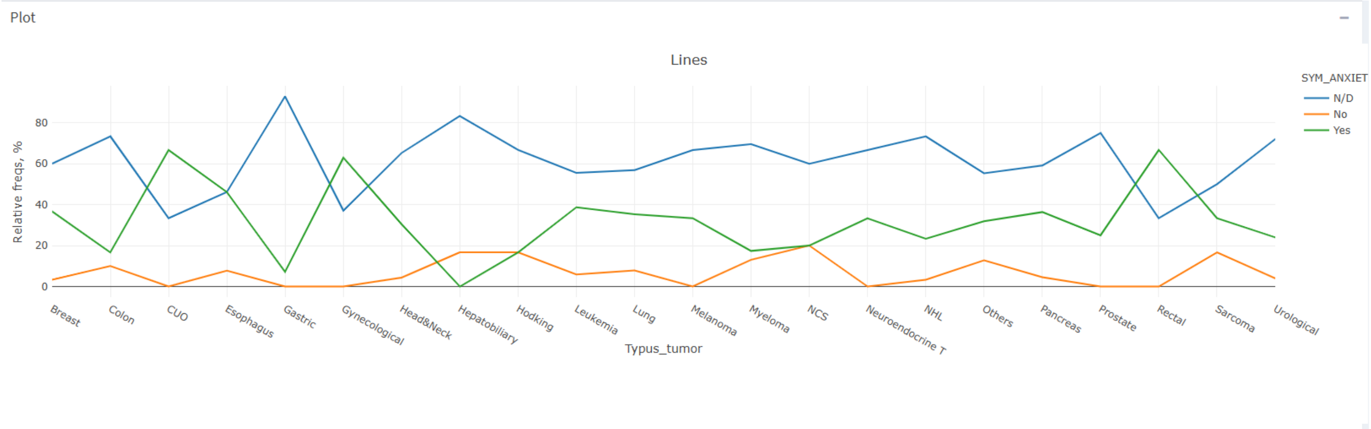

Statistics

SYM_ANXIETY	Breast	Colon	CUO	Esophagus	Gastric	Gynecological	Head&Neck	Hepatobiliary	Hodking	Leukemia	Lung	Melanoma	Myeloma	NCS	Neuroendocrine T	NHL	Others	Pancreas	Prostate	Rectal	Sarcoma	Urological
N/D	60	73.3	33.3	46.2	92.9	37	65.2	83.3	66.7	55.5	56.9	66.7	69.6	60	66.7	73.3	55.3	59.1	75	33.3	50	72
No	3.3	10	0	7.7	0	0	4.3	16.7	16.7	5.9	7.8	0	13	20	0	3.3	12.8	4.5	0	0	16.7	4
Yes	36.7	16.7	66.7	46.2	7.1	63	30.4	0	16.7	38.7	35.3	33.3	17.4	20	33.3	23.3	31.9	36.4	25	66.7	33.3	24

b) Registry of Anxiety & tumor types & PCP=Yes

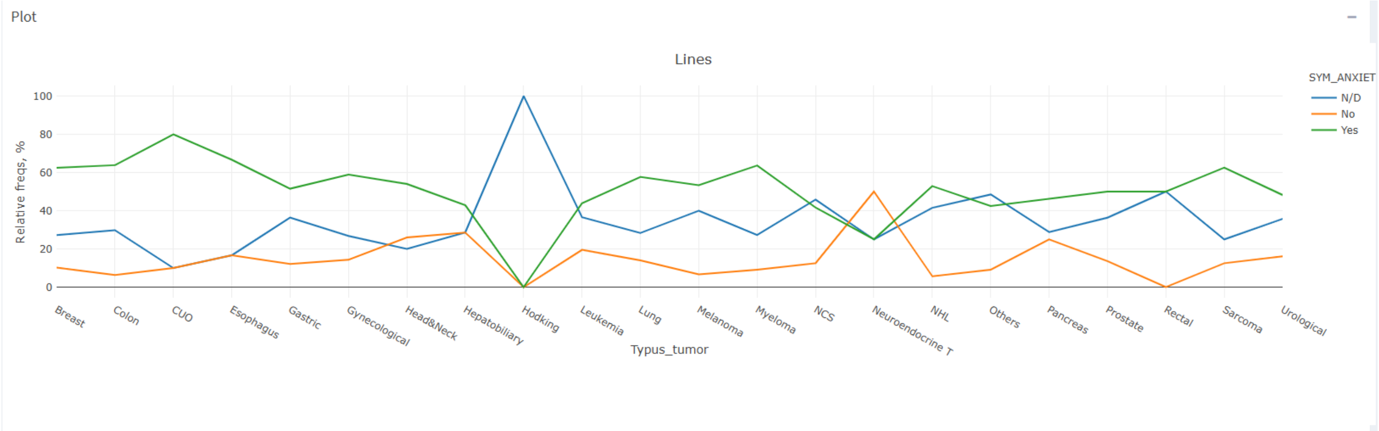

Statistics

SYM_ANXIETY	Breast	Colon	CUO	Esophagus	Gastric	Gynecological	Head&Neck	Hepatobiliary	Hodking	Leukemia	Lung	Melanoma	Myeloma	NCS	Neuroendocrine T	NHL	Others	Pancreas	Prostate	Rectal	Sarcoma	Urological
N/D	27.3	29.8	10	16.7	36.4	26.8	20	28.6	100	36.6	28.3	40	27.3	45.8	25	41.5	48.5	28.8	36.4	50	25	35.7
No	10.2	6.4	10	16.7	12.1	14.3	26	28.6	0	19.5	14	6.7	9.1	12.5	50	5.7	9.1	25	13.6	0	12.5	16.1
Yes	62.5	63.8	80	66.7	51.5	58.9	54	42.9	0	43.9	57.6	53.3	63.6	41.7	25	52.8	42.4	46.2	50	50	62.5	48.2

Note: N/D (in blue): not registered data. Yes (in green) registered data and the symptoms are positive. No (in orange) registered data bad the symptom is negative. Anxiety in a) was registered in 34.6.% and register was positive in 29.8%; in b) was registered in 68.7%, and register was positive in 34.1%. Statistical difference between groups a) & b): p<0.001 (see table 1)

In the group "Yes-PCP" (patients included in Palliative care program), the global register is double than in de "Non-PCP" group, and the positive records is upper to.

In the case of Hodgkin's disease, there are only two cases, which is why the curve produces an abrupt inflection.

Figure S4. Time curves (survival at 100 days from advanced disease) comparison by support care programs and groups Yes/No.

a) Time curves (at 100 d) for Palliative Care Program (PCP) groups

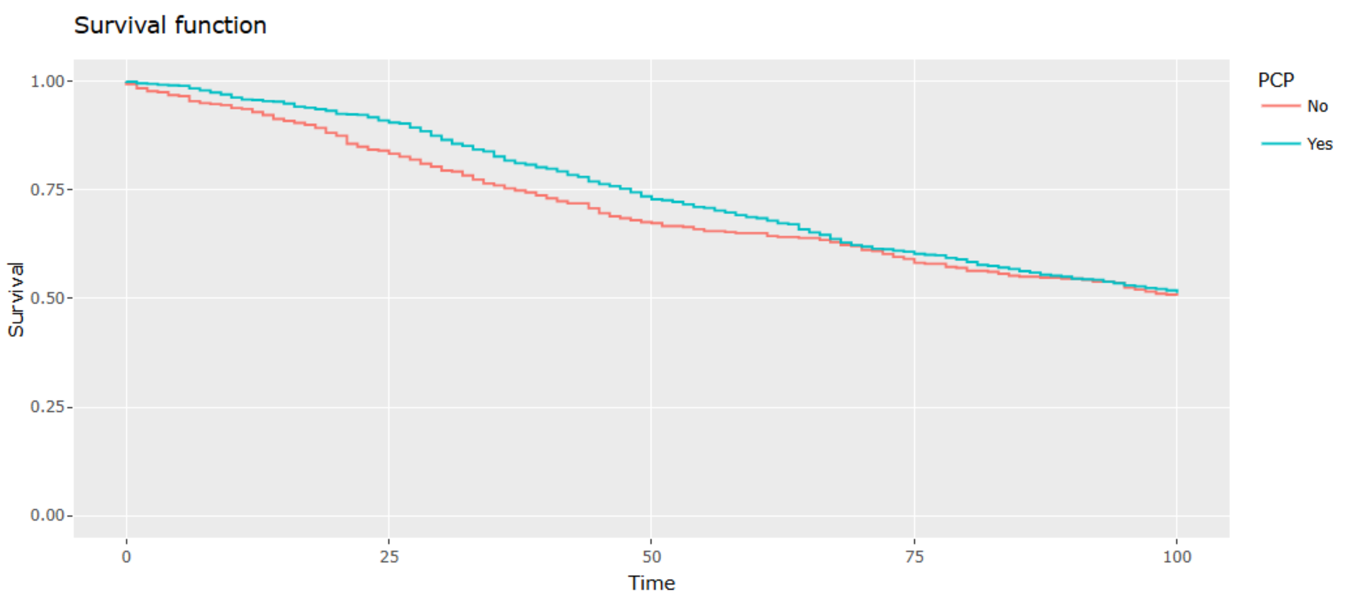

Group	N	Observed (O)	Expected (E)	$(O-E)^2/E$	$(O-E)^2/V$
PCP=No	438	216	206.86	0.4	0.6
PCP=Yes	854	416	425.14	0.2	0.6

χ^2= 0.60 on 1 degrees of freedom
p-value =0.996

b) Time curves (at 100d) for Psico-social Program (PSOP) groups

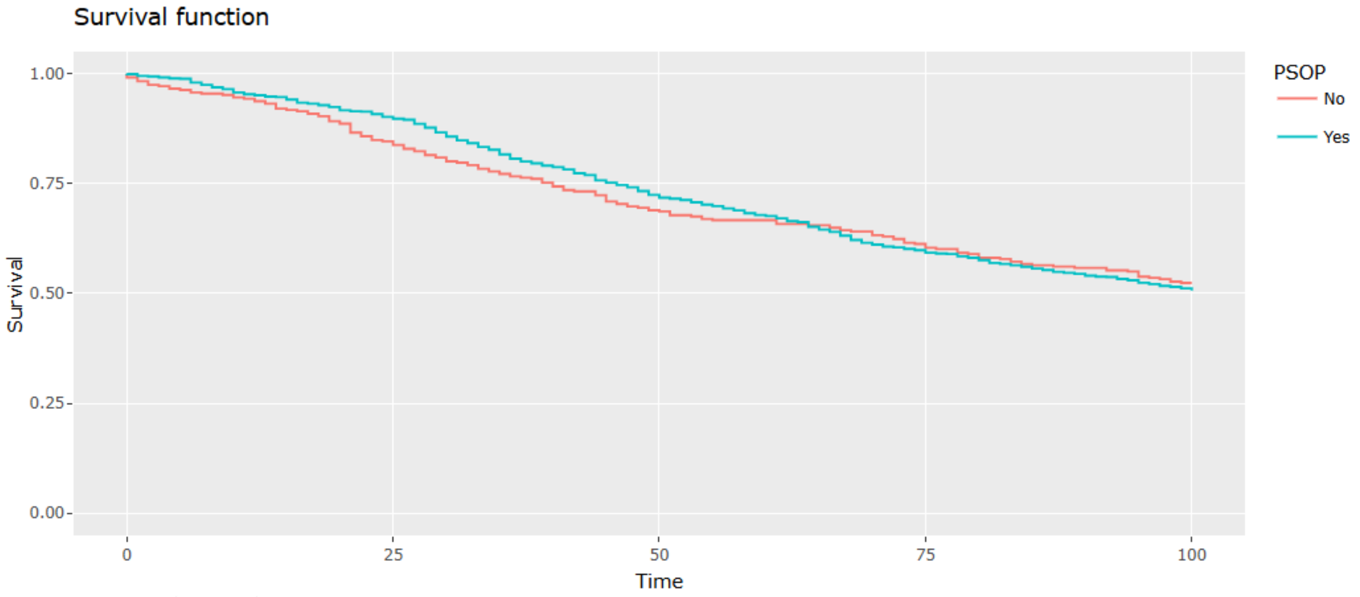

Group	N	Observed (O)	Expected (E)	$(O-E)^2/E$	$(O-E)^2/V$
PSOP=No	351	167	168.51	0.01	0.02
PSOP=Yes	936	462	460.49	0.00	0.02

χ^2= 0.02 on 1 degrees of freedom
p-value =1.000

Figure S5. Time curves (survival at 100 days from advanced disease) comparison by Prognostics factors.

a) Time curves (at 100d AD) for ECOG

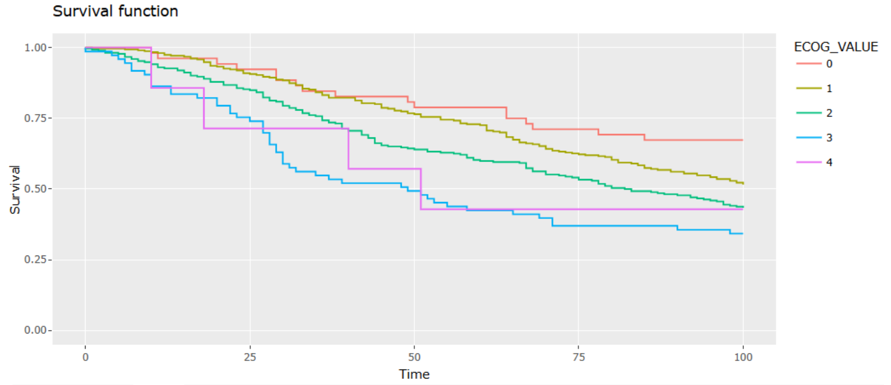

Group	N	Observed (O)	Expected (E)	(O-E)²/E	(O-E)²/V
ECOG_VALUE=0	52	17	31.09	6.39	7.02
ECOG_VALUE=1	310	150	174.20	3.36	6.36
ECOG_VALUE=2	272	154	135.69	2.47	3.91
ECOG_VALUE=3	73	48	29.00	12.45	13.62
ECOG_VALUE=4	7	4	3.02	0.32	0.32

χ^2= 25.22 on 4 degrees of freedom

p-value <0.001

b) Time curves (at 100d AD) for Therapeutic regimen

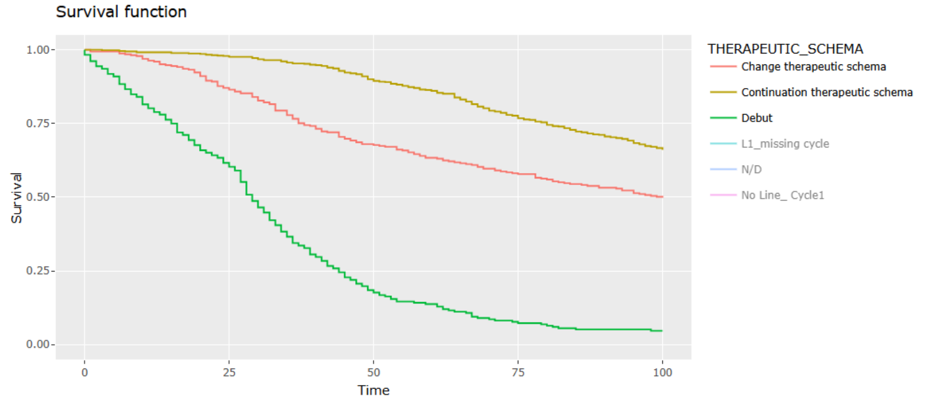

Group	N	Observed (O)	Expected (E)	(O-E)²/E	(O-E)²/V
THERAPEUTIC_SCHEMA=Change therapeutic schema	325	163	154.69	0.45	0.60
THERAPEUTIC_SCHEMA=Continuation therapeutic schema	711	241	416.01	73.62	219.63
THERAPEUTIC_SCHEMA=Debut	232	221	47.48	634.13	716.41
THERAPEUTIC_SCHEMA=L1_missing cycle	10	3	6.05	1.53	1.56
THERAPEUTIC_SCHEMA=N/D	11	3	6.26	1.70	1.73
THERAPEUTIC_SCHEMA=No Line_ Cycle1	3	1	1.52	0.18	0.18

χ^2= 744.95 on 5 degrees of freedom

p-value =0.000

c) Time curves (at 100d AD) for SACT Trial

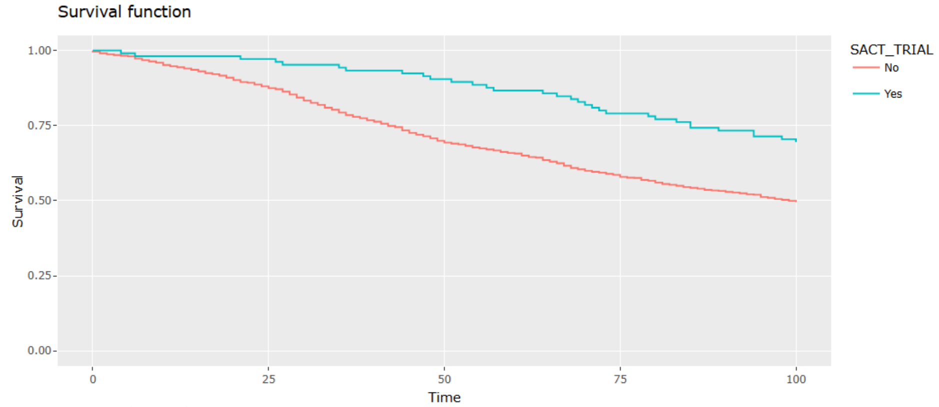

Group	N	Observed (O)	Expected (E)	(O-E)²/E	(O-E)²/V
SACT_TRIAL=No	1181	596	566.31	1.56	15.97
SACT_TRIAL=Yes	105	32	61.69	14.29	15.97

χ^2= 15.97 on 1 degrees of freedom

p-value =0.014

Note: in the first two prognostic factors (a: ECOG and b: therapeutic regimen) statistically significant differences are shown. Regarding clinical trials, in c, this statistical difference has a p-value=0.014S7 Time curves (survival at 100 days from advanced disease) comparison by Prognostics factors, but for each pathology group: Solid Tumor & Hematological Neoplasm

Figure S6. Time curves comparison (survival at 100 days of advanced disease) by prognostic factors, but for each pathology group: Solid Tumor and Hematological Neoplasia.

**Solid Tumor (ST)**	**Hematological Neoplasm (HN)**

a.i) Time curves (at 100d AD) for ECOG Group ST

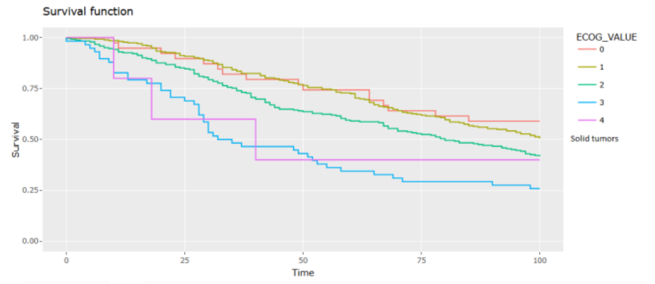

Group	N	Observed (O)	Expected (E)	$(O-E)^2/E$	$(O-E)^2/V$
ECOG_VALUE=0	39	16	23.57	2.43	2.63
ECOG_VALUE=1	273	135	163.33	4.91	9.59
ECOG_VALUE=2	242	141	127.47	1.44	2.32
ECOG_VALUE=3	58	43	21.59	21.22	22.90
ECOG_VALUE=4	5	3	2.03	0.46	0.47

χ^2= 30.80 on 4 degrees of freedom
p-value <0.001

a.ii) Time curves for ECOG: loses significance p=0.561

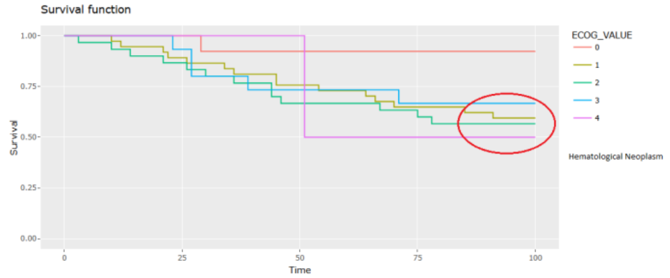

Group	N	Observed (O)	Expected (E)	$(O-E)^2/E$	$(O-E)^2/V$
ECOG_VALUE=0	13	1	5.48	3.66	4.37
ECOG_VALUE=1	37	15	13.24	0.23	0.38
ECOG_VALUE=2	30	13	10.13	0.82	1.15
ECOG_VALUE=3	15	5	5.42	0.03	0.04
ECOG_VALUE=4	2	1	0.74	0.09	0.09

χ^2= 4.87 on 4 degrees of freedom
p-value =0.561

b.i) Time curves (at 100d AD): Therapeutic regimen Group ST

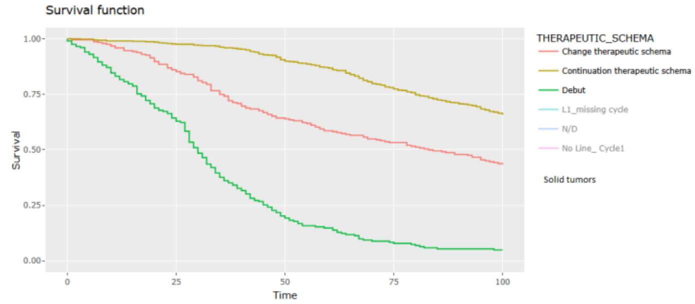

Group	N	Observed (O)	Expected (E)	$(O-E)^2/E$	$(O-E)^2/V$
THERAPEUTIC_SCHEMA=Change therapeutic schema	244	138	114.88	4.65	5.94
THERAPEUTIC_SCHEMA=Continuation therapeutic schema	615	210	376.87	73.88	243.42
THERAPEUTIC_SCHEMA=Debut	202	192	43.62	504.78	574.08
THERAPEUTIC_SCHEMA=L1_missing cycle	9	3	5.61	1.21	1.23
THERAPEUTIC_SCHEMA=N/D	9	3	5.15	0.90	0.91
THERAPEUTIC_SCHEMA=No Line_ Cycle1	2	1	0.88	0.02	0.02

χ^2= 614.67 on 5 degrees of freedom
p-value =0.000

b.ii) Time curves for Therapeutic regimen: no differences between continuation or change regimen

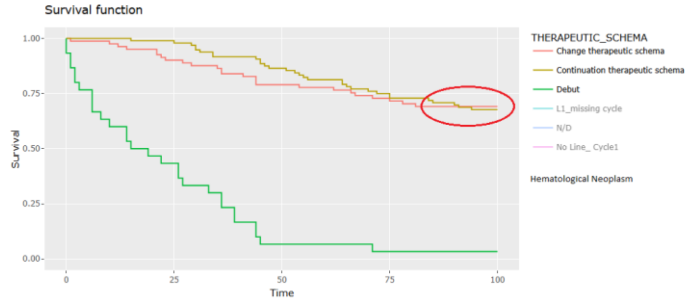

Group	N	Observed (O)	Expected (E)	$(O-E)^2/E$	$(O-E)^2/V$
THERAPEUTIC_SCHEMA=Change therapeutic schema	81	25	34.88	2.80	4.78
THERAPEUTIC_SCHEMA=Continuation therapeutic schema	96	31	43.69	3.69	7.66
THERAPEUTIC_SCHEMA=Debut	30	29	4.38	138.23	151.77
THERAPEUTIC_SCHEMA=L1_missing cycle	1	0	0.51	0.51	0.52
THERAPEUTIC_SCHEMA=N/D	2	0	1.02	1.02	1.04
THERAPEUTIC_SCHEMA=No Line_ Cycle1	1	0	0.51	0.51	0.52

χ^2= 152.87 on 5 degrees of freedom
p-value =0.000

c.i) Time curves (at 100d AD) for SACT Trial. Group ST

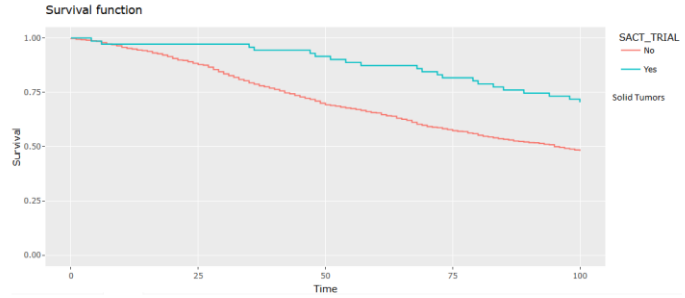

Group	N	Observed (O)	Expected (E)	$(O-E)^2/E$	$(O-E)^2/V$
SACT_TRIAL=No	1006	523	499.85	1.07	13.32
SACT_TRIAL=Yes	71	21	44.15	12.14	13.32

χ^2= 13.32 on 1 degrees of freedom
p-value =0.038

c.ii) Time curves for SACT Trial. Group HN

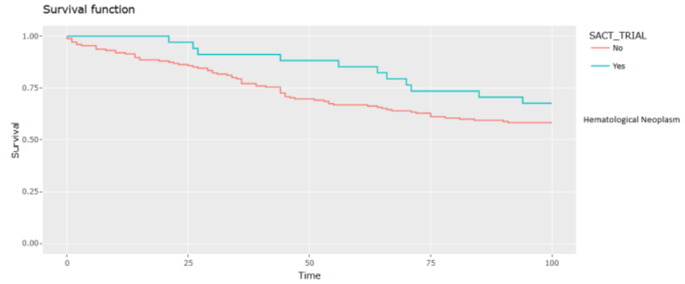

Group	N	Observed (O)	Expected (E)	$(O-E)^2/E$	$(O-E)^2/V$
SACT_TRIAL=No	175	73	68.59	0.28	1.56
SACT_TRIAL=Yes	34	11	15.41	1.26	1.56

χ^2= 1.56 on 1 degrees of freedom
p-value =0.956

Note: In the first two prognostic factors (a.i) ECOG and b.i) therapeutic regimen) the Solid Tumor group maintains the statistically significant differences, but the Hematological Neoplasia group lose for ECOG (a.ii) & for Therapeutic regimen (b.ii): no differences between continuation or change of regimen, and it is maintain with debuts. Regarding clinical trials, in c.i & c.ii, the statistical differences are not significant, has a value of p=0.038 for ST and p=0.956 for HN.

Figure S7. Shows the number of bags of blood transfuses by tumor types.

Plot

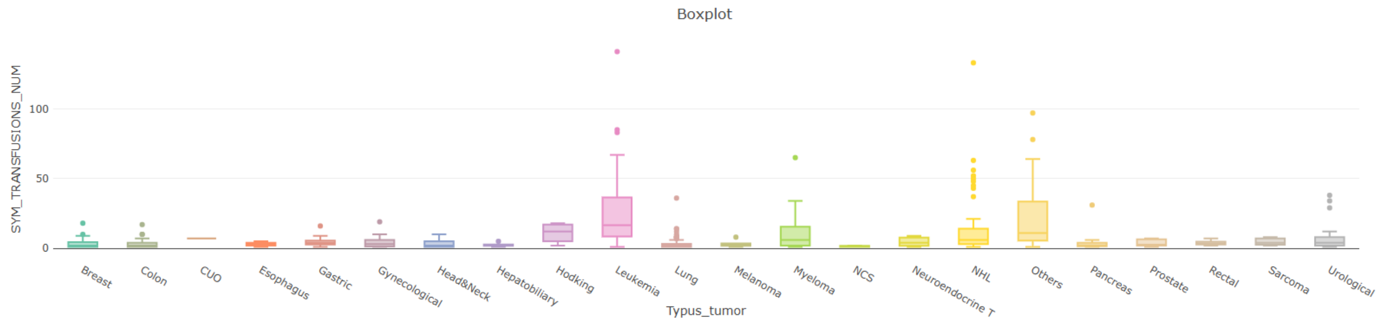

Statistics

Typus_tumor	Mean	SD	Median	Q1	Q3	N
Breast	3.375000	3.6345120	2.0	1.00	4.25	32
Colon	3.538461	3.8076845	2.0	1.00	4.00	26
CUO	7.000000	NA	7.0	7.00	7.00	1
Esophagus	2.900000	1.1972190	3.0	2.00	3.75	10
Gastric	4.647059	3.7406747	4.0	3.00	5.00	17
Gynecological	4.148148	3.9389935	3.0	1.50	6.00	27
Head&Neck	3.090909	2.8268962	2.0	1.00	5.00	22
Hepatobiliary	2.400000	1.5165751	2.0	2.00	2.00	5
Hodking	11.000000	7.3936910	12.0	6.50	16.50	4
Leukemia	25.881579	24.6743954	16.5	8.75	34.75	76
Lung	3.109489	3.8053346	2.0	1.00	3.00	137
Melanoma	3.000000	2.8284271	2.0	2.00	2.00	5
Myeloma	10.689655	14.0537322	6.0	2.00	15.00	29
NCS	1.333333	0.5773503	1.0	1.00	1.50	3
Neuroendocrine T	4.666667	4.0414519	4.0	2.50	6.50	3
NHL	13.270270	20.1734243	6.0	3.00	14.00	74
Others	21.181818	23.8661038	11.0	6.00	32.00	33
Pancreas	3.958333	5.9889693	2.0	1.75	4.00	24
Prostate	3.625000	2.5599944	2.0	2.00	6.25	8
Rectal	3.800000	1.9235384	3.0	3.00	4.00	5
Sarcoma	4.666667	3.0550505	4.0	3.00	6.00	3
Urological	7.884615	10.0849469	4.0	2.00	7.75	26

Legend: SD: standard deviation / Q1: first quartile or 25th percentile / Q3: third quartile or 75th percentile / N: number of available values / (*Neuroendocrine tumors: only with 2 patients). The greatest consumption of bags of blood products is observed in hematological pathologies. The "Others" group includes myelodysplastic syndromes (MDS) and lymphoproliferative syndromes (LPS).
